# Protein phosphatase 2 regulatory subunit B''Alpha silencing inhibits tumor cell proliferation in liver cancer

**DOI:** 10.1002/cam4.2620

**Published:** 2019-10-24

**Authors:** Huijuan Chen, Jing Xu, Peixiao Wang, Qingming Shu, Lihong Huang, Jing Guo, Xuyi Zhang, Hongying Zhang, Ying Wang, Zhongyang Shen, Xinguo Chen, Qing Zhang

**Affiliations:** ^1^ Department of Liver Transplantation The Third Medical Centre Chinese PLA (People's Liberation Army) General Hospital Beijing China; ^2^ Graduate School Anhui Medical University Hefei China; ^3^ National Translational Science Center for Molecular Medicine & Department of Cell Biology State Key Laboratory of Cancer Biology Fourth Military Medical University Xi'an China; ^4^ Department of Gastroenterology Henan Children's Hospital Zhengzhou China; ^5^ Pathology Department The Third Medical Centre Chinese PLA (People's Liberation Army) General Hospital Beijing China; ^6^ Medical Department The Third Medical Centre Chinese PLA (People's Liberation Army) General Hospital Beijing China; ^7^ Department of Transplantation Surgery Tianjin First Central Hospital Tianjin China

**Keywords:** liver cancer, migration, PPP2R3A, proliferation

## Abstract

**Aim:**

To explore the effects of protein phosphatase 2 regulatory subunit B''Alpha (*PPP2R3A*) on the proliferation and migration of liver cancer cells.

**Methods:**

Expression of PPP2R3A in tumor tissues of hepatocellular carcinoma (HCC) patients was detected by immunohistochemistry and western blotting. In two liver cancer cell lines (HepG2 and HuH7), PPP2R3A expression was silenced and then overexpression with PPP2R3A lentiviral vectors, and the effects of PPP2R3A knockdown or overexpression on the proliferation, cell cycle, migration, and invasion of HCC cells were determined in vitro. In a xenograft cancer model in nude mice, the in vivo effects of PPP2R3A knockdown on tumor growth and cancer cell proliferation were evaluated.

**Results:**

PPP2R3A expression was found in tumor foci in six of eight HCC samples, at a level higher than that in the adjacent para‐tumor tissues. PPP2R3A expression was observed primarily in the cytoplasm of the cancer cells. Knockdown of PPP2R3A resulted in significant inhibition of hepatoma cell proliferation (*P* < .05), migration (*P* < .01), and invasion (*P* < .01) as well as a significant delay in the G1/S transition in both liver cancer lines (*P* < .05) and increased p53 expression. Conversely, overexpression of PPP2R3A promoted the proliferation (*P* < .05) and altered cell cycle progression (*P* < .05) of both liver cancer cell lines. In vivo, PPP2R3A knockdown in liver cancer cells led to significant reductions in the tumor volume (*P* < .001) and the expression of Ki‐67 in tumor tissues (*P* < .05).

**Conclusion:**

PPP2R3A may play a role in liver cancer via the regulation of tumor cell proliferation and invasion.

## Graphical abstract

The PPP2R3A may play a pro‐tumor function in HCC by regulating the proliferation and invasion of HCC cells.

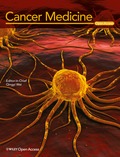

## 1 INTRODUCTION

Liver cancer ranks sixth among the most common cancers worldwide, with more than 850 000 new cases diagnosed each year, at a ratio of about five males to one female.^1^ It is the fourth leading cause of cancer‐related mortality in the world,^2^ and the annual mortality rate in China is as high as 55%.^3^ Hepatocellular carcinoma (HCC) is the most common primary malignancy in the liver, accounting for 85%–90% of primary liver cancer cases. The oncogenic alterations of gene function, due to gene mutations (such as in *TERT*, *TP53*, and *CTNNB1*), epigenetic changes, or altered transcriptional regulation can lead to the tumorigenesis of liver cancer and therefore represent potential drug targets for cancer therapy. However, the effective biotherapeutic drugs for liver cancer have remained elusive. The molecular and genetic mechanisms underlying the liver cancer tumorigenesis need to be better understood to facilitate the development of more effective treatments.

Protein phosphatase 2A (PP2A) is the major cellular Ser/Thr protein phosphatase that regulates multiple cellular processes, including metabolism, DNA repair, proliferation, cell motility, apoptosis, and autophagy.^4^, ^5^, ^6^, ^7^, ^8^, ^9^ Moreover, PP2A is known to be involved in tumorigenesis.^10^, ^11^, ^12^, ^13^ Early studies identified PP2A as a tumor suppressor, but recent studies supported an oncogenic function of PP2A. These contradictory findings might be explained by the diversity of its structure.^14^, ^15^ The holoenzyme of PP2A is a heterotrimeric structure,^10^ composed of a structural subunit A (Aα or Aβ), a catalytic subunit C (Cα or Cβ), and a regulatory subunit B, which is encoded by a set of different genes classified as B/PR55, B'/PR61, B", and B'" families.^16^, ^17^ The regulatory subunit B determines the substrate specificity and the holoenzyme subcellular localization, suggesting that referring simply to the function of PP2A provides insufficient biological relevance. The functions of the various regulatory subunits, which are likely cellular context‐specific and may or may not depend on the enzyme activity, must to be investigated in detail.

Protein Phosphatase 2 Regulatory Subunit B"Alpha (PPP2R3A), also known as PR72/B"α2 or PR130/B"α1,^18^ belongs to the PP2A regulatory subunit B" family and has been found to regulate several important cancer‐related signaling pathways, such as the Wnt‐signaling cascade, epidermal growth factor (EGF)‐EGF receptor (EGFR) signaling, and the 5′ adenosine monophosphate‐activated protein kinase (AMPK) activity.^19^, ^20^, ^21^, ^22^ In the recent years, these signaling pathways have been found to play essential roles, either via abnormal activation or inactivation, in the occurrence and development of liver cancer, through such functions as regulating cell adhesion ability, promoting an inflammatory environment, and enhancing cell proliferation, migration, and invasion.^23^, ^24^, ^25^, ^26^, ^27^, ^28^, ^29^, ^30^ These findings imply a potential role for PPP2R3A in liver cancer, but considering its cellular context‐specificity, the function of PPP2R3A in liver cancer remains to be elucidated. In the present study, we investigated the effect of PPP2R3A on the malignancy of liver cancer cells in vitro and in vivo, and our results provide evidence for the potential of PPP2R3A as a novel drug target for liver cancer therapy.

## 2 MATERIALS AND METHODS

### 2.1 Liver cancer specimen collection and immunohistochemical staining

Liver cancer specimens were collected at the General Hospital of Chinese People's Armed Police Forces. All cases were histologically confirmed to be primary HCC. This study was approved by the Ethics Committee of the General Hospital of the Chinese People's Armed Police Force. All procedures involving human participants were performed in accordance with the ethical standards of the institutional and national research committee and with the 1964 Declaration of Helsinki and its later amendments or comparable ethical standards. Written informed consent was obtained from all individual participants included in the study.

Immunohistochemical staining was performed as described previously^31^ on serial 4‐μm thick sections prepared from each tissue block. The primary antibodies used for staining were the anti‐PPP2R3A (Sigma‐Aldrich, 1:200 dilution) and anti‐Ki67 antibody (ZSGB‐BIO, not diluted).

For the Ki67 proliferation assay, five random fields were photographed and the total number of proliferating liver cancer cells is counted using Image‐pro Plus to calculate the positive score for Ki67 staining. The Ki‐67 expression levels were scored according to the criteria of the International Ki67 Working Group.^32^

### 2.2 Western blotting analysis

Western blot analysis was performed as described previously.^33^ Briefly, human liver cancer tissue specimens were lysed in tissue lysate buffer (radioimmunoprecipitation assay [RIPA]: phenylmethylsulfonyl fluoride [PMSF] = 100:1, Sigma‐Aldrich). Total protein from the tissue lysates or cell lysates was quantified and then run on 8%–10% sodium dodecyl sulfate polyacrylamide gel electrophoresis and blotted onto polyvinylidene difluoride (PVDF) membranes (Sigma‐Aldrich). The blots were probed with primary antibody: anti‐PPP2R3A (Sigma‐Aldrich, 1:500 dilution, rabbit source) or anti‐p53 (Cell Signaling Technology, 1:500 dilution, mouse source) overnight at 4°C. After washing, the sections were then incubated with secondary antibody (ZSGB‐BIO, 1:50 000 dilution). The chemiluminescent signals were visualized using ECL‐Spray (Advansta) and a chemiluminescence imaging analysis system (Tanon). β‐actin (Sigma‐Aldrich, 1:500 dilution, rabbit source), tubulin (Cell Signaling Technology, 1:5000 dilution, rabbit source), and glyceraldehyde‐3‐phosphate dehydrogenase (GAPDH; Cell Signaling Technology, 1:5000 dilution, mouse source) were used as the internal controls.

### 2.3 Cell culture and virus infection

The human liver cancer cell line Huh‐7 was purchased from the JCRB Cell Bank, and the HepG2 cell line was obtained from the Institute of Cell Biology, Chinese Academy of Sciences. Cells were cultured in Dulbecco's Modified Eagle's Medium (DMEM) supplemented with 10% fetal bovine serum (FBS, Gibco) at 37°C in a humidified incubator of 5% CO_2_ (Thermo Fisher Scientific).

Two PPP2R3A short hairpin RNA (shRNA) lentiviral vectors (shRNA‐PPP2R3A‐6328/shRNA1 and shRNA‐PPP2R3A‐6332/shRNA2) and the corresponding negative control vector (shRNA‐3NC), a lentiviral vector (PPP2R3A) for overexpression of PPP2R3A, and the corresponding negative control vector (5NC) were purchased from GenePharma. These viral vectors contained the green fluorescent protein (GFP) expression sequence. Liver cancer cells were seeded in six‐well plates at a density of 3 × 10^5^ cells per well and infected with the above lentiviral vectors, separately, according to the manufacturer's instructions. Then the intensity of green fluorescence expression by the cells was observed. After the infected cells were screened with puromycin (4 μg/mL) (Sigma‐Aldrich) for 2 weeks, they were used for further assays.

### 2.4 Quantitative real‐time polymerase chain reaction (qRT‐PCR) analysis

Total RNA was extracted from the cultured cells using the Total RNA Kit II (Omega Bio‐Tek). The RNA samples with an A260/A280 ratio between 1.9 and 2.1 were selected and reversely transcribed to cDNA using the Prime Script^TM^ RT reagent kit (TaKaRa Bio). Next, they were quantified using the SYBR Premix EX TaqⅡ (TaKaRa Bio). The following PCR primers were used: hPPP2R3A, forward 5′‐AGAGAAGACAGGATTTGTGACAGCA‐3′ and reverse 5′‐CAGTTGGGCTTTGCTAGAAGACAG‐3′; hACTB, forward 5′‐CGTGGACATCCGCAAAGA‐3′ and reverse 5′‐GAAGGTGGACAGCGAGGC‐3′. The RT‐PCR assays were performed with the MJ Mini Detection System (Bio‐Rad, Hercules). The relative expression of mRNA was calculated according to the 2^−ΔΔCt^ method. hACTB was used as an internal reference.

### 2.5 Cell proliferation assay

Cell proliferation was analyzed using the Cell Counting Kit‐8 reagent (CCK‐8, Engreen). Liver cancer cells were seeded (1 × 10^3^/well in 100‐μL medium) in 96‐well plates containing DMEM with 10% FBS. After continuous incubation for different times (24h, 48h, 72h, 96h or 120 h), 10 μL of CCK‐8 solution was added to each well during this time period. The absorbance of the solution in each well at a wavelength of 450 nm was measured using a microplate reader (Bio‐Rad).

### 2.6 Cell cycle assay

A cell cycle assay was performed using the Cell Cycle Detection Kit (KeyGEN BIOTECH). First, the cells were trypsinized with 0.25% trypsin, washed twice with ice‐cold phosphate‐buffered saline (PBS), and then fixed in 70% ethanol at 4°C overnight. After the addition of 100 μL of RNase A, the cells were incubated in a 37°C water bath for 30 minutes and then 400 μL of propidium iodide (PI) was added for staining at 4°C for 30 minutes in darkness. Samples were analyzed using a FAC Scan flow cytometer (Becton‐Dickinson) and the red fluorescence was recorded at the excitation wavelength of 488 nm.

### 2.7 Cell migration assay

The cell migration ability was evaluated using 24‐well Millicell chambers (BD Biosciences). Millicell chambers were placed in a sterile 24‐well plate. The cells were digested, washed with PBS, and resuspended in DMEM containing 1% bovine serum albumin (BSA, Sigma‐Aldrich). Then, the cells (5 × 10^4^/well, 200 μL) were seeded into the upper chamber, and 600‐μL DMEM containing 10% FBS was added to the lower chamber. After culture in 5% CO_2_ within a 37°C incubator for 48 hours, the chambers were removed and swabs were used to remove the cells in the upper chamber. The chamber membranes were washed in PBS and fixed in 4% paraformaldehyde (Sigma‐Aldrich) for 20 minutes, followed by air drying. The chamber membranes were stained with 0.1% crystal violet for 10 minutes and washed with flowing water. After air drying, five random fields were selected and photographed from every group using a light microscope.

### 2.8 Transwell invasion assay

The Matrigel invasion assay was performed using Millicell chambers (BD Biosciences). After the frozen Matrigel (BD Biosciences,) was thawed at 4°C overnight, the membrane of each chamber was coated with diluted Matrigel (1:6, 50 μL/cm^2^) and incubated at 37°C for 2 hours to allow formation of a matrix barrier. The cells (1 × 10^5^/well) were seeded into the upper chamber in serum‐free medium, and the lower chamber was filled with 600‐μL medium containing 10% FBS. After the cells were cultured at 37°C for 48 hours, the cells on the upper membrane surface were removed, and the cells that passed through the pores were fixed with 4% paraformaldehyde for 20 minutes. Subsequently, the cells were stained with 0.1% crystal violet for 10 minutes and then washed with flowing water. After air drying, five random fields were selected and photographed from every group using a light microscope.

### 2.9 Xenograft tumor model in nude mice

The BALB/c nude mice (6‐8 weeks) were purchased from Spewford Biotechnology Co., Ltd and raised in the Animal Center of the Beijing Academy of Military Medical Sciences. Animal studies were approved by the local regulatory agency (Institutional Animal Care and Use Committee of Laboratory Animal Center of Academy of Military Medical Sciences).

Nude mice were randomly divided into three groups, namely the shRNA1, shRNA2, and shRNA‐3NC groups, with six mice per group. After the cells were digested, they were resuspended in serum‐free DMEM and then mixed with Matrigel (ratio 1:1) and stored at 4°C. Then 150 μL of the cells (1 × 10^7^/mL) were injected into the dorsal portions of the nude mice. The weight of the nude mice and the size of the tumor were measured from the third week after the inoculation and then every 3 days using a digital caliper. The tumor volume was calculated using the formula: *V* (volume) = (*π* × length × width^2^)/6. The tumor tissues were removed after 30 days, immediately fixed in formalin, and then examined using hematoxylin & eosin (H&E) staining and immunohistochemistry.

### 2.10 Statistical analysis

All data are presented as mean ± standard deviation (SD) from at least three independent experiments. We applied SPSS 18.0 (SPSS Inc) to perform the data analysis and applied GraphPad Prism 5.0 to draw the graphs. Quantitative variables were analyzed between two groups using Student's *t* test or among multiple groups using one‐way analysis of variance (ANOVA). *P* values of <0.05 were considered statistically significant.

## 3 RESULTS

### 3.1 Expression of PPP2R3A in HCC tissues

**Figure 1:**
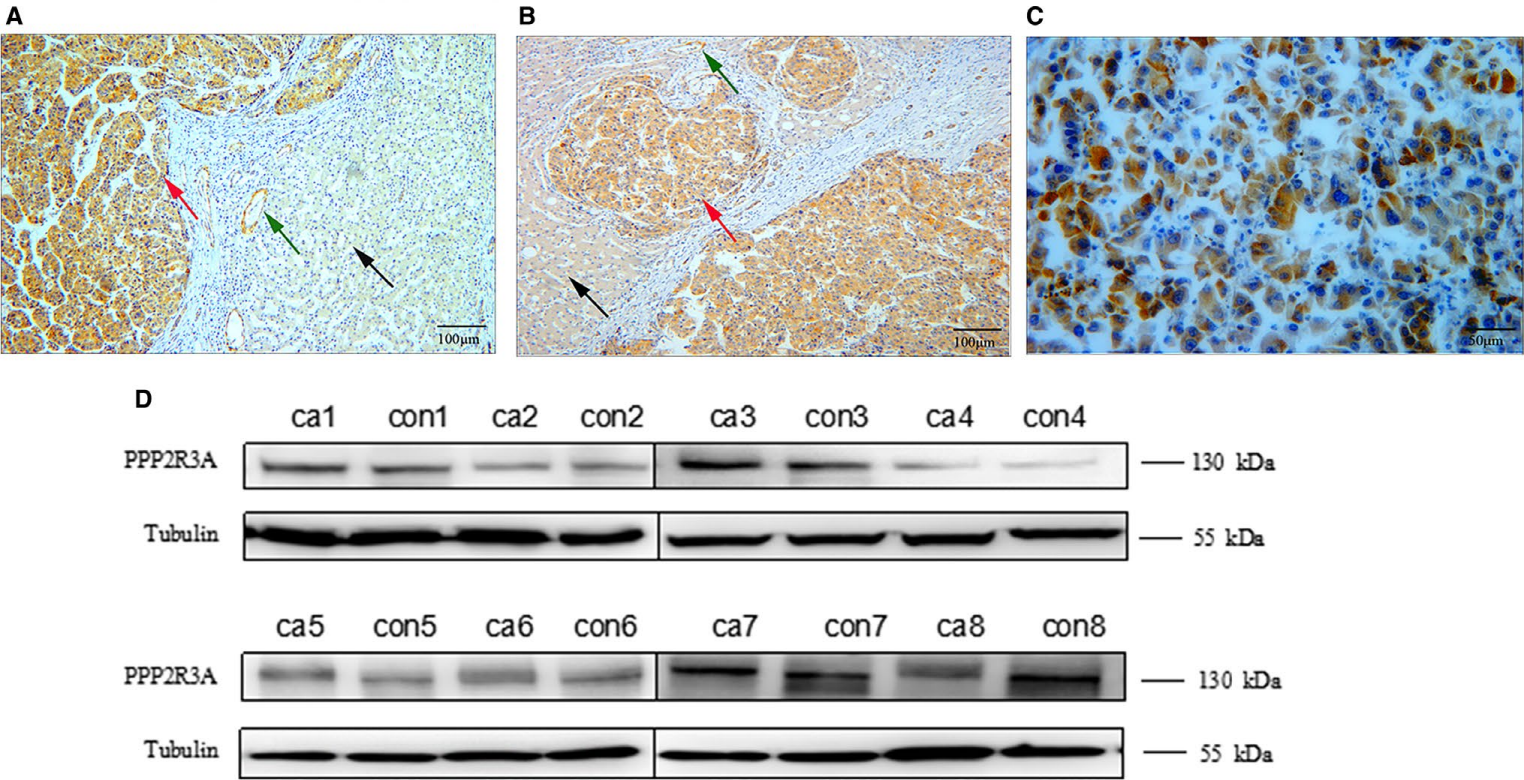
Expression of PPP2R3A in tumor tissues of hepatocellular carcinoma (HCC) patients. Within liver cancer specimens, we searched for evidence of PPP2R3A expression in the HCC cells (red arrow), endothelial cells (green arrow), and adjacent para‐tumor tissues (black arrow) via immunohistochemical staining. n = 8. A, PPP2R3A staining was strongly positive in cancerous tissues but negative in the adjacent para‐tumor liver tissues. The representative images were taken under a light microscopy at a magnification of 100× (scale bar, 100 μm). B, In another representative image, strong positive staining for PPP2R3A expression is seen in cancerous tissues while only weak positive staining for PPP2R3A expression is seen in adjacent tissues, (magnification, 100×;scale bar, 100 μm). C, Strong staining of PPP2R3A was detected mainly in the cytoplasm of HCC cells. The representative images of the tumor foci were taken under a light microscopy at a magnification of 200× (scale bar, 50 μm). D, Protein expression of PPP2R3A in the liver cancer tissues from eight HCC patients, as detected by western blotting. ca, tumor tissue; con, the paired adjacent para‐tumor tissue

To explore the potential role of PPP2R3A in liver cancer, we examined the expression of PPP2R3A via immunohistochemical staining in eight liver cancer tissue specimens from HCC patients. Positive PPP2R3A expression was found in HCC cells in six of eight specimens, while negative or very low‐level PPP2R3A expression was observed in the liver cells in the adjacent para‐tumor tissues (Figure 1A,B). In these cancerous lesions stained with the anti‐PPP2R3A antibody, a diffuse and strong pattern was observed in four specimens, and a partial pattern in two specimens. In addition, the positive staining of PPP2R3A was detected mainly in the cytoplasm of HCC cells (Figure 1C) and sporadically in the endothelial cells in the stroma adjacent to cancer lesions (Figure 1A,B).

Western blotting analysis of the tissue lysates also showed a higher expression level of PPP2R3A in tumor foci than in the adjacent para‐tumor tissues in six of eight HCC patients (Figure 1D), which was consistent with the results of immunohistochemical analysis.

### 3.2 Gene knockdown of PPP2R3A in liver cancer cells

**Figure 2:**
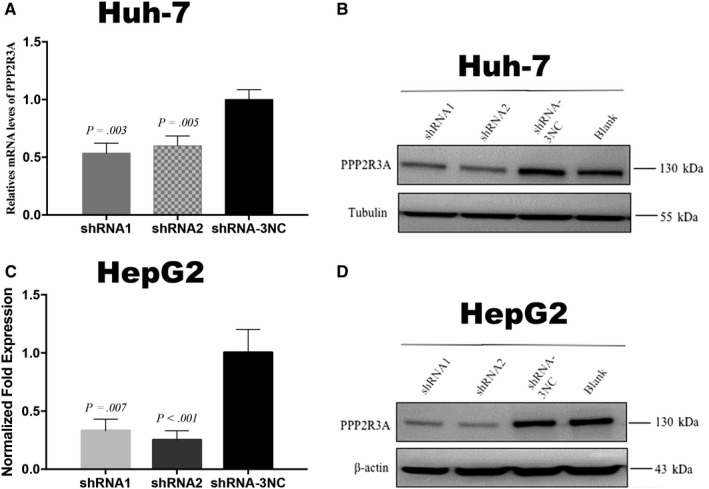
Efficacy of shRNA‐PPP2R3A (shRNA1 and shRNA2) for knockdown of PPP2R3A expression in liver cancer cells. PPP2R3A mRNA levels were measured by qRT‐PCR in Huh‐7 Cells (A) and HepG2 cells (C). PPP2R3A protein expression was detected by western blotting assay in Huh‐7 Cells (B) and HepG2 Cells (D)

To explore the effect of *PPP2R3A* gene knockdown on the malignant behaviors of liver cancer cells, we constructed two shRNA lentiviral vectors, namely shRNA‐PPP2R3A‐6328 (shRNA1) and shRNA‐PPP2R3A‐6332 (shRNA2), to infect two liver cancer cell lines, Huh‐7 and HepG2, individually. A scramble shRNA lentiviral vector, shRNA‐3NC, was used as the negative control. After 48 hours of virus infection, fluorescence microscopy revealed that the infection rates of the two liver cancer cells were both above 90% (data not shown), and the knockdown effect on PPP2R3A expression was detected by qRT‐PCR and western blotting. In the Huh‐7 and HepG2 cells, the expression level of PPP2R3A was significantly knocked down by the two shRNA vectors both at the mRNA (*P* < .01 or *P* < .001; Figure 2A,C) and protein levels (Figure 2B,D), compared with that by the negative control vector.

### 3.3 Knockdown of PPP2R3A inhibits cell proliferation in liver cancer cells

**Figure 3:**
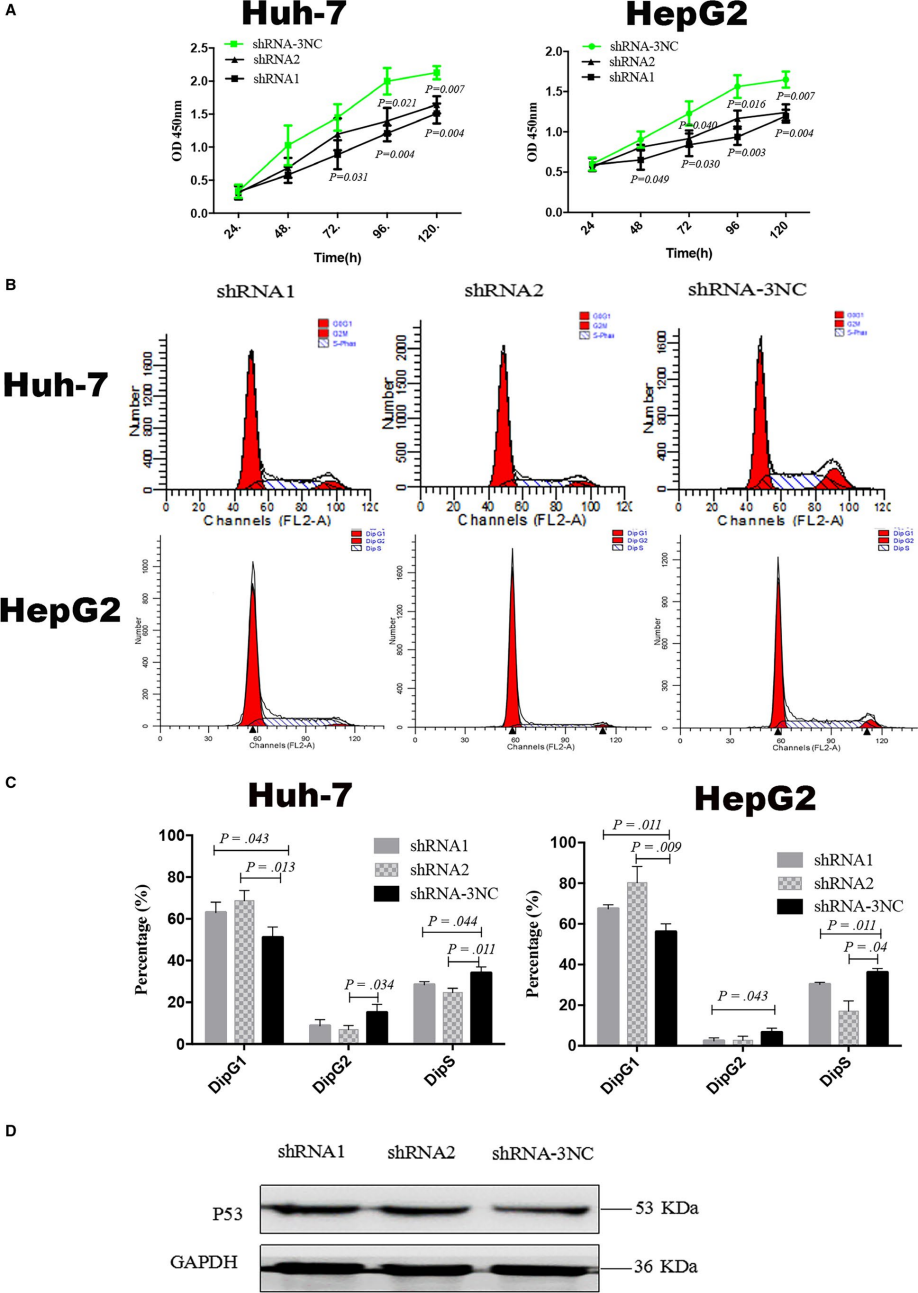
Knockdown of PPP2R3A inhibits liver cancer cell proliferation and G1/S transition. A, The proliferation of Huh‐7 (left) and HepG2 (right) cells after PPP2R3A knockdown was detected using the CCK‐8 assay. B, The cell cycle of distributions of Huh‐7 and HepG2 cells was analyzed by flow cytometry after propidium iodide (PI) staining. C, Statistical analysis of the percentages of Huh‐7 (left) and HepG2 (right) cells in the G1, S, and G2 phases. D, p53 protein expression in the liver cancer cells was detected by western blotting

Malignant proliferation is the predominant hallmark of cancer cells. Here we used the CCK‐8 assay to detect the effects of PPP2R3A knockdown on the proliferation of liver cancer cells. The results showed that at 48 hours after PPP2R3A knockdown, the proliferation of liver cancer cells was inhibited (*P* < .05) compared with that of control cells, and this difference in the proliferation rate continued to increase with more time in culture (*P* < .01; Figure 3A). To analyze cell cycle control progression following PPP2R3A knockdown in liver cancer cells, we detected the DNA content of the cells via flow cytometry after PI staining. The results showed that PPP2R3A knockdown resulted in an obvious shift in the cell cycle of liver cancer cells (Figure 3B), with their arrest in G1/S phase. Accordingly, the percentage of liver cancer cells in G1 phase was significantly increased after PPP2R3A knockdown, while that in S phase was significantly decreased (*P* < .05, *P* < .01; Figure 3C). In addition, via western blotting analysis, we found that PPP2R3A knockdown in liver cancer cells increased the level of endogenous p53 (Figure 3D). These results demonstrated that knockdown of PPP2R3A in liver cancer cells inhibited cell proliferation, led to an arrest in G1/S phase, and upregulated the expression of p53, which plays a major role in the G1/S checkpoint.

### 3.4 Knockdown of PPP2R3A inhibits the migration and invasion of liver cancer cells

**Figure 4:**
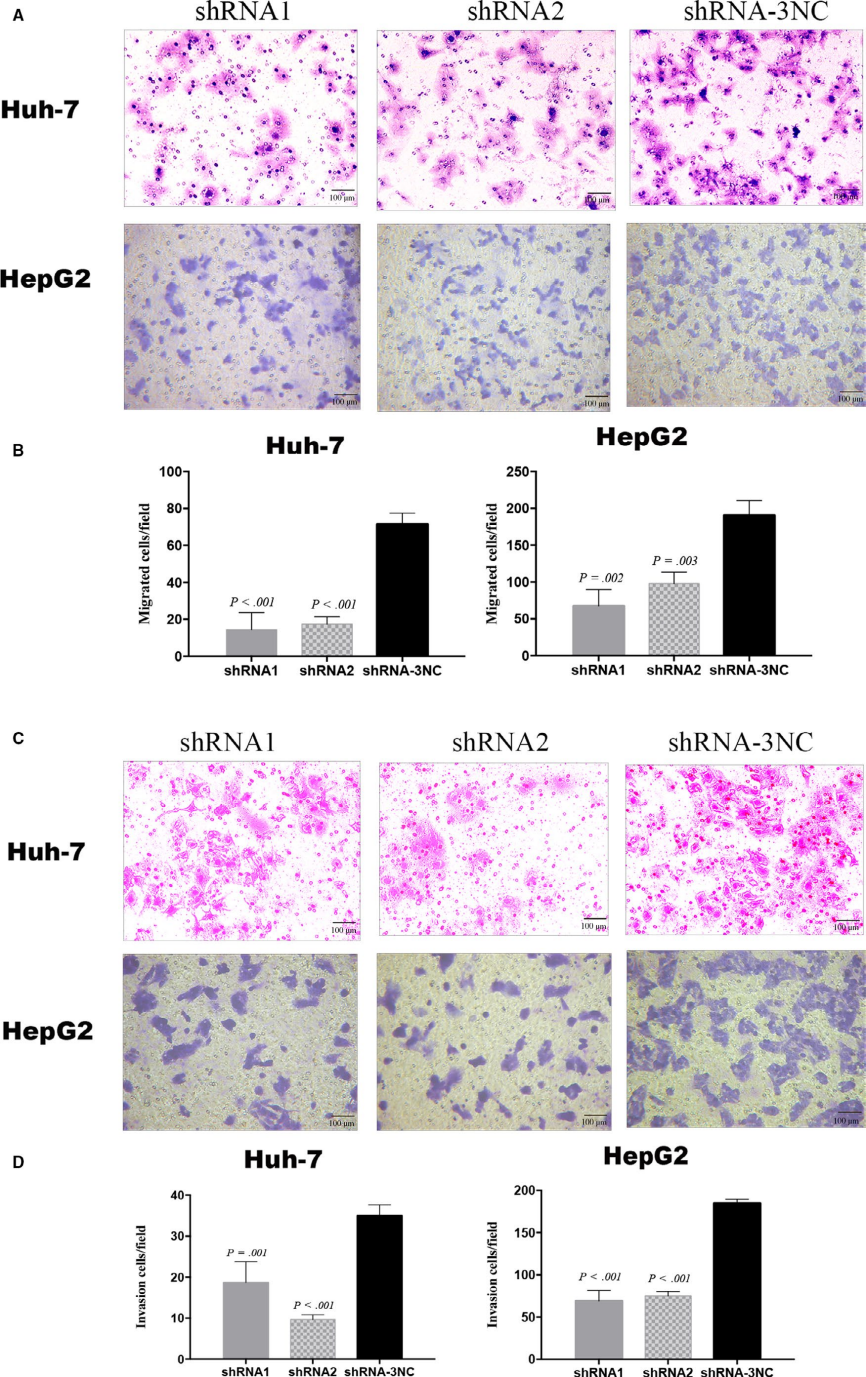
Downregulation of PPP2R3A inhibits liver cancer cell migration and invasion. A, The migration potential of Huh‐7 and HepG2 cells was examined in a Transwell assay, and representative images of the migrated liver cancer cells are shown. Scale bar = 50 μm. B, Statistical analysis of the numbers of Huh‐7 (left) and HepG2 (right) cells that had migrated across the chamber membrane. C, The invasive ability of Huh‐7 and HepG2 cells was assessed in a Transwell assay employing Matrigel‐coated Transwell chambers. Representative images of the invading liver cancer cells are shown. Scale bar = 50 μm. D, Statistical analysis of Huh‐7 (left) and HepG2 (right) cells invasion across the chamber membrane

Using a Transwell assay, we assessed the effects of PPP2R3A knockdown on migratory and invasive potential of liver cancer cells. PPP2R3A knockdown led to significant reduction in the numbers of Huh‐7 (*P* < .001) and HepG2 (*P* < .01) cells crossing the chamber membrane (Figure 4A,B), compared with the numbers of migrating cells in the negative control group. Specifically, after infection with shRNA1 or shRNA2 for PPP2R3A knockdown, the numbers of migrating Huh‐7 cells decreased by 80.5%±11.0% and 75.4%±7.6%, respectively, and the numbers of migrating HepG2 cells decreased by 63.5%±16.5% and 48.0%±10.1%, respectively. Similarly, PPP2R3A knockdown significantly decreased the number of cells that passed through Matrigel in the chamber invasion experiments (*P* < .01;Figure 4C,D). After infection with shRNA1 or shRNA2, 47.1% ± 11.7% and 72.1% ± 5.6% respective reductions in Huh‐7 cell invasion as well as 62.6% ± 6.2% and 59.2% ± 3.8% respective reductions in HepG2 cells invasion were observed. These results showed that PPP2R3A knockdown inhibited the invasive potential of liver cancer cells, possibly via the suppression of the cell migration.

### 3.5 Knockdown of PPP2R3A inhibits the tumor growth and liver cancer cell proliferation in vivo

**Figure 5:**
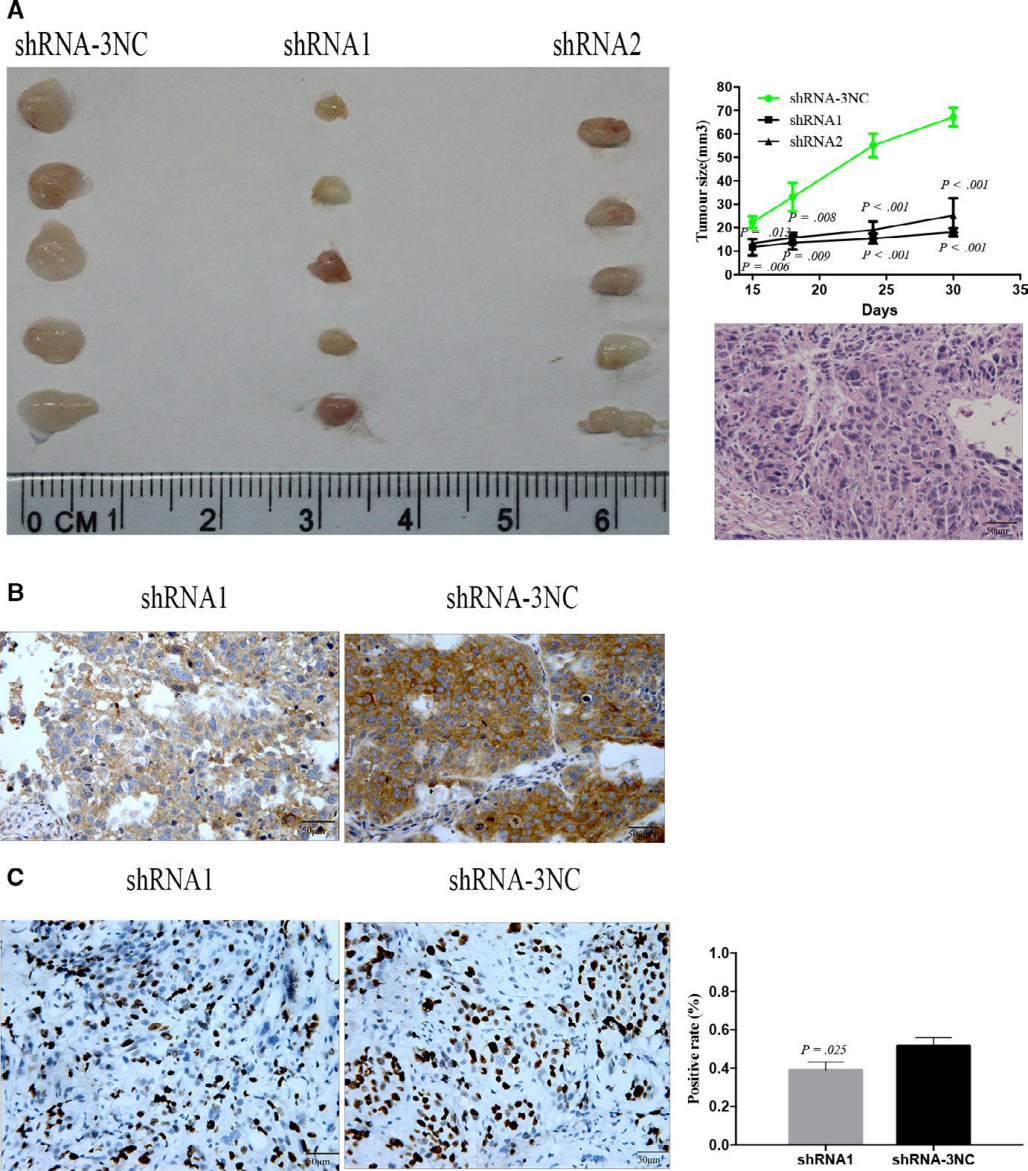
Downregulation of PPP2R3A in liver cancer cells inhibits tumor growth in nude mice. A, Comparisons of tumor size in nude mice 30 d after inoculation of PPP2R3A‐deficient or control liver cancer cells, as well as tumor volume based on measurements taken every 3 d until the mice were sacrificed. n = 5. Representative images of H&E staining of the tumor tissues from the nude mice are shown in panel A. Scale bar = 50 μm. B, The location of PPP2R3A expression in the tumor tissues of the mice inoculated with shRNA1‐ (left) and shRNA‐3NC‐ (right) treated cells was detected by immunohistochemical staining. The representative image was taken under light microscopy at a magnification of 200×. Scale bar = 50 μm. C, Ki‐67 expression was detected by immunohistochemical staining in tumor tissues rom mice inoculated with shRNA1‐ (left) and shRNA‐3NC‐ (right) treated cells. The Ki‐67‐positive rate within the tumor tissues from nude mice was statistically analyzed

To detect whether downregulation of PPP2R3A in liver cancer cells influences tumor growth in vivo, we constructed a xenograft tumor model in nude mice. PPP2R3A knockdown (shRNA1 or shRNA2) in Huh‐7 cells significantly slowed the tumor growth in vivo, compared with that seen in the negative control group (*P* < .001) (Figure 5A). H&E staining revealed the morphological characteristics of Huh‐7 cells in the tumor tissues. In addition, we evaluated PPP2R3A expression in the tumor tissues and, as expected, found that PPP2R3A expression was obviously lower in the tumors formed by cells with PPP2R3A knockdown than in those formed by the control Huh‐7 cells (Figure 5B), confirming the knockdown efficacy of PPP2R3A in liver cancer cells in vivo. These data suggested that downregulation of PPP2R3A could effectively inhibit liver cancer tumor growth in vivo.

Next, we analyzed the effect of PPP2R3A knockdown on the in vivo proliferative potential of liver cancer cells based on the detection of Ki‐67 expression in tumor tissues harvested from the nude mouse models. We found that Ki67 labeling index for tumor cells was significantly lower in the PPP2R3A knockdown (shRNA1) group than in the control group (*P* = .025; Figure 5C), demonstrating that the knockdown of PPP2R3A inhibited the proliferation of the liver cancer cells in vivo.

### 3.6 Overexpression of PPP2R3A promotes proliferation of liver cancer cells

**Figure 6:**
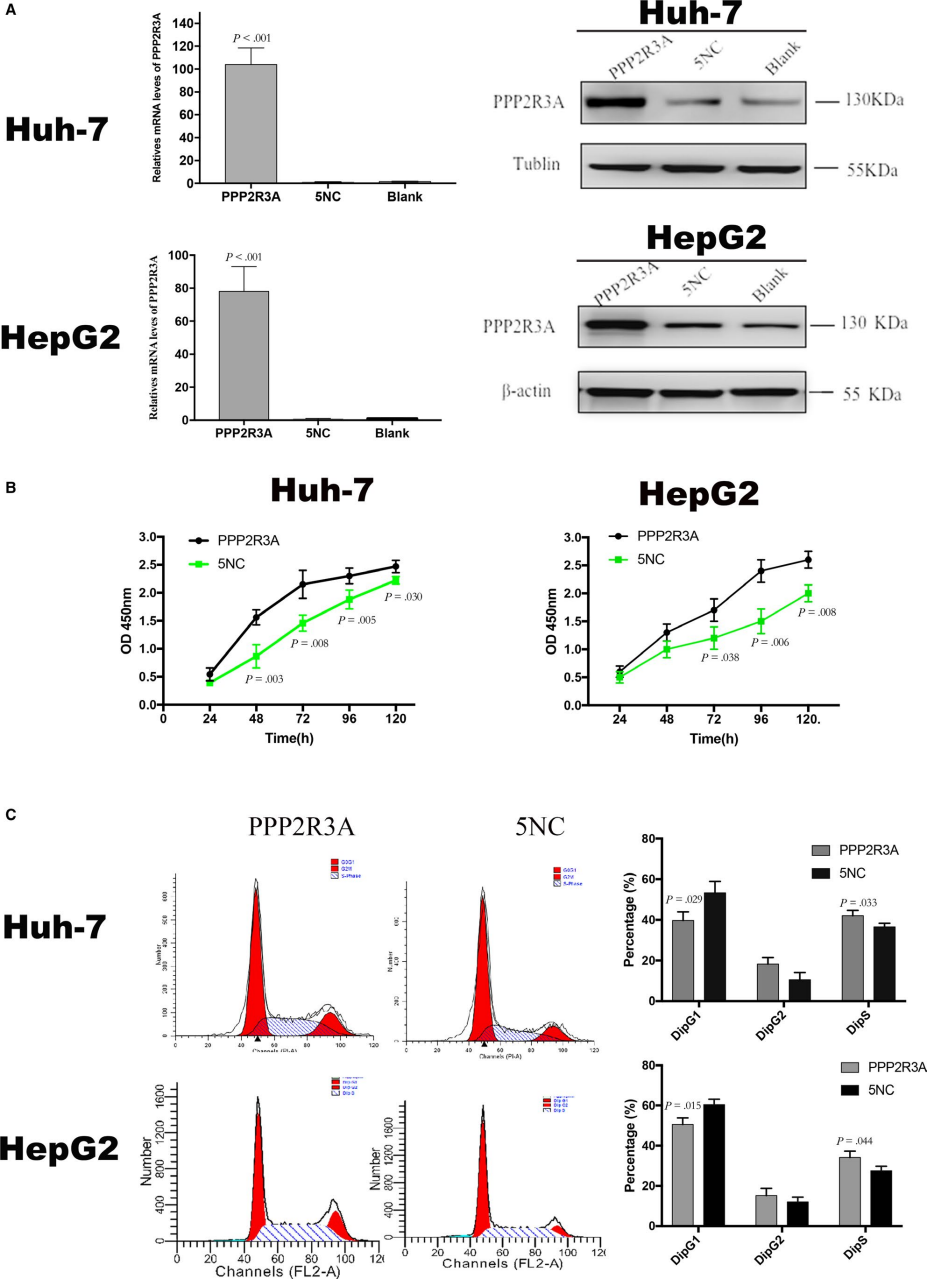
Overexpression of PPP2R3A promotes proliferation and G1/S transformation of hepatocellular carcinoma cells. A, PPP2R3A overexpression in Huh‐7 (upper) and HepG2 (lower) cells after lentiviral infection was confirmed by qRT‐PCR (left) and western blotting (right). B, The proliferation of Huh‐7 (left) and HepG2 (right) cells was detected by CCK‐8 assay. C, The cell cycle distribution of Huh‐7 and HepG2 cells was analyzed by flow cytometry after PI staining (left), and the percentages of Huh‐7 and HepG2 cells in G1, S, and G2 phases were statistically analyzed (right)

To further confirm the role of PPP2R3A in the malignant behavior of liver cancer cells, we constructed a lentiviral vector for overexpression of PPP2R3A and infected Huh‐7 and HepG2 cells. After 48 hours of virus infection, fluorescence microscopy showed that the infection rates for both liver cancer cell types were higher than 90%. Moreover, PPP2R3A overexpression in both cell lines was confirmed by qRT‐PCR and western blotting analyses, in comparison with PPP2R3A expression in cells treated with the negative control vector (*P* < .001; Figure 6A). Using the CCK‐8 assay, we found that PPP2R3A overexpression promoted liver cancer cell proliferation. After 48 hours in culture following viral infection, a difference in the proliferation rates of liver cancer cells overexpressing PPP2R3A and the control cells appeared, with the cells overexpressing PPP2R3A exhibiting the higher proliferation rates (*P* < .05; Figure 6B). Additionally, we analyzed the cell cycle distribution of the two cell types with or without PPP2R3A overexpression by flow cytometry. The results showed that overexpression of PPP2R3A significantly changed the cell cycle distribution of liver cancer cells compared with the respective control cells (Figure 6C). In contrast to the results obtained with PPP2R3A knockdown in liver cancer cells, the percentage of liver cancer cells in G1 phase was significantly decreased, while that in S phase was significantly increased (both *P* < .05).

## 4 DISCUSSION

In the present study, we provide the first evidence of PPP2R3A protein expression in liver cancer and demonstrate its promoting effects on the proliferation and migration of liver cancer cells in vitro and in vivo. We found that PPP2R3A protein expression was higher in tumor tissues than in adjacent para‐tumor tissues of HCC patients. In vitro, knockdown of PPP2R3A significantly inhibited liver cancer cell proliferation, while overexpression of PPP2R3A promoted liver cancer cell proliferation. Moreover, both knockdown and overexpression of PPP2R3A led to shifts in the cell cycle distribution, with opposing effects on the percentages of cells in G1/S phases. PPP2R3A knockdown also significantly suppressed the migration and invasive potential of HCC cells. Together, our results suggest that PPP2R3A may be associated with the occurrence of liver cancer via the regulation of tumor cell proliferation and invasion.

Our staining experiment to detect PPP2R3A expression in liver cancer tissues showed that PPP2R3A protein was mainly located in the cytoplasm of HCC cells and partially expressed on the cell membrane. Moreover, PPP2R3A protein expression was high in HCC tissues, but not in the adjacent para‐tumor tissues. This differential expression in HCC suggested that PPP2R3A may play a biological role in liver cancer. PPP2R3A belongs to the regulatory subunit B" family of PP2A, an important member of the serine/threonine phosphatase family. PPP2R3A encodes two splice variants, PR130 and PR72. Previous studies have reported that the PPP2R3A subtype PR130 can redistribute SHIP2 (SH2 domain‐containing phosphatidylinositol‐3,4,5‐trisphosphate 5‐phosphatase) to the cell membrane to prevent EGF‐induced EGFR degradation.^19^ Therefore, the cytoplasm and membrane may be the sites of the biological function of PPP2R3A in liver cancer.

PP2A regulates many cellular processes and plays an important role in the progression of many tumors, including the proliferation of tumor cells.^34^ Previous studies showed that PPP2R3A is involved in the regulation of PP2A activity as well as some tumor‐associated signaling pathways, such as EGF/EGFR, β‐catenin, etc^19^, ^35^ Specifically, PPP2R3A (PR130) was found to prevent EGF‐induced EGFR degradation, sustaining EGF‐mediated signaling and possibly supporting cell proliferation based on the tumor growth‐promoting effects of EGFR in various tumors.^19^ In our present study, knockdown of PPP2R3A using shRNA lentiviral vectors significantly inhibited the proliferation of liver cancer cells in vitro and also led to significantly less tumor growth compared with the control group in the xenograft tumor model. Based on the observed effects of PPP2R3A knockdown on tumor cell growth in vitro and in vivo, we propose that PPP2R3A acts to enhance the proliferation of liver cancer cells. Additionally, the expression rate of Ki‐67 is positively correlated with the degree of tumor malignancy,^36^ and in our xenograft model, the rate of Ki‐67 expression in tumor tissues formed from PPP2R3A‐deficient cells was significantly lower than that of tumor tissues formed from control liver cancer cells, further suggesting that PPP2R3A enhances the malignant behavior of tumor cells by promoting their proliferation.

p53 protein is frequently mutated in a variety of cancer types, and its abnormal expression is closely associated with increased tumor cell proliferation and changes in the cell cycle distribution of tumor cells. In normal cells, p53 levels are low, as the protein is degraded via the ubiquitin‐proteasome system. However, abnormal expression of p53 protein can affect the proliferation of tumor cells.^37^, ^38^ Here, we found that knockdown of PPP2R3A caused upregulation of p53 expression, suggesting that PPP2R3A may affect the proliferation of liver cancer cells via its regulation of p53 expression.

From another perspective, PP2A can target Cdc6 via PR70 and promote dephosphorylation in a calcium‐modulating manner to inhibit cell cycle progression.^39^ PPP2R3A has a structural core similar to PR70 and may affect the cell cycle in the same way.^40^ In a very recent study, Göder et al found that loss of PPP2R3A (PR130) expression results in the early arrest of S phase^41^ and that ablation of PR130 can reduce the dephosphorylation of ataxia telangiectasia mutated (ATM). Phosphorylated ATM activates the downstream CHK1, CHK2, and p53 genes, thereby inducing cell cycle arrest. We also found that knockdown of PPP2R3A induced arrest of cells in the G1/S phase, while overexpression of PPP2R3A significantly promoted conversion of G1/S phase. These results suggest that PPP2R3A might play a role in regulating the cell cycle of liver cancer cells. However, additional research is needed to determine whether p53 is indirectly involved in the PPP2R3A regulation of the cell cycle in liver cancer.

Liver cancer is a malignant tumor with strong invasive ability. Previous studies have found that PR130 plays a role in the migration of HT1080 fibrosarcoma cells by influencing focal adhesion formation.^42^ Our data showed that PPP2R3A silencing did not only obviously inhibited migration of liver cancer cells, but did significantly suppress the invasive potential of liver cancer cells. These preliminary findings together with our results demonstrating the expression of PPP2R3A in the tumor tissues of liver cancer patients suggest that PPP2R3A may be a promising target for liver cancer therapy.

The present study has some limitations. Although we observed that knockdown or overexpression of PPP2R3A can affect the proliferation of liver cancer cells by affecting cell cycle progression, the related mechanism remains unclear. Additional research is also needed to determine which cyclins are involved and whether they affect the downstream pathway of p53 protein. Further research exploring the specific mechanism by which PPP2R3A affects the cell cycle progression and proliferation of liver cancer cells is required.

In conclusion, our present study showed that PPP2R3A plays an important role in the proliferation and invasion of liver cancer cells and may be a potential drug target for liver cancer therapy. The mechanism of PPP2R3A's role in liver cancer warrants further attention.

## Data Availability

The datasets generated during and analysed during the current study are available from the corresponding author on reasonable request.
